# Behavioral profile, lifestyle and social skills in Portuguese adolescents

**DOI:** 10.1186/s12889-021-10355-1

**Published:** 2021-02-18

**Authors:** Clarisse Magalhães, Maria Fátima Ribeiro, Maria Raquel Esteves, Luísa Aires, Sara Lima, Gustavo Silva, Assunção Nogueira, Teresa Herdeiro, Susana Pedras

**Affiliations:** 1 CESPU, Institute of Research and Advanced Training in Health Sciences and Technologies, Rua Central de Gandra, 1317, 4585-116 Gandra, PRD Portugal; 2 University Institute of Maia (ISMAI), Castelo da Maia, Portugal; 3 Research Center in Physical Activity, Health and Leisure (CIAFEL), Porto, Portugal; 4 High School Augusto Gomes , Matosinhos, Porto Portugal; 5 Research Center in Sports Sciences, Health and Human Development (CIDESD), Maia, Portugal; 6 Department of Medical Sciences, Institute of Biomedicine – iBiMED, University of Aveiro, Aveiro, Portugal

**Keywords:** Behavioral profile, Lifestyle, Social skills, Adolescents

## Abstract

**Background:**

Seventy percent of premature deaths in adults are due to behaviors initiated during adolescence. Therefore, it is essential to promote individual and social behaviors that educate adolescents in the ability to make healthy choices. Accordingly, the main goals of this study were to characterize Lifestyles and Social Skills, as well as identify homogeneous subgroups, in a sample of Portuguese adolescents.

**Methods:**

A cross-sectional study was conducted, including 1008 adolescents attending the 7th to the 9th grades of five middle schools from the Tâmega and Sousa regions of Portugal, and using the *My Lifestyle Questionnaire* and the *Social Skills Inventory*. To establish a profile of the participants, a Cluster Analysis (K-means) was performed, and the Jaccard coefficient was used to assess the stability of the solution found.

**Results:**

From the total sample, 556 adolescents with a mean age of 13.43 years (SD = 1.1) were included in the analysis. The majority of the sample presented a healthy lifestyle (72.26%) and 50.7% of the adolescents had a highly elaborated repertoire of Social Skills. Moreover, three clusters were found. Cluster 1 (*n* = 92) showed a less elaborate repertoire of Social Skills and was designated as the “Adjusted”. Cluster 2 (*n* = 115) comprised adolescents with a good repertoire of Social Skills and was named the “Sociable”. Cluster 3 (*n* = 258) was composed of adolescents with a highly elaborate repertoire of Social Skills and the best Lifestyle indicators and was named the “Healthy”.

**Conclusions:**

The group of adolescents in the cluster called the “Sociable” needs to be included in health education and Social Skills programs. Nutrition and Monitored Safety behaviors reveal low values and, therefore, present a greater need for awareness, sensitization, and intervention in the school context. For this reason, the promotion of a healthy lifestyle should be part of the academic curriculum and transversal to all academic disciplines.

**Supplementary Information:**

The online version contains supplementary material available at 10.1186/s12889-021-10355-1.

## Background

The leading causes of illness and deaths among young adults are largely preventable [1] and studies have shown that about 70% of premature adult deaths are due to behaviors initiated during adolescence (e.g. tobacco use, drugs, and reckless driving) [2]. Therefore, any effort to understand and improve the knowledge about health and risk behaviors in adolescents is warranted and welcomed.

In adolescence, the most studied lifestyle behaviors are nutrition and physical activity. These two have shown to be commonly associated, mainly because obesity is a chronic disease related to an unbalanced diet and low levels of physical activity [3–5], with a worrying prevalence in Portugal, especially in the group of adolescents between 10 and 12 years old, where 23.1% are overweight and 9.6% are obese [6].

Tobacco and alcohol consumption, sleep patterns, and protective sexual behaviors are also important targets of studies and interventions [7–10]. The behaviors associated with road safety, which prevent injuries and accidents, are also important and include avoiding traveling with someone who has drunk too much, driving within speed limits, and wearing seat belts. Some studies have also suggested a relationship between road accidents, alcohol, and substance consumption [10, 11]. Besides, the use of drugs and medicines not prescribed by a physician, and the use of sedatives or amphetamines are closely associated with adverse consequences and with unhealthy growth patterns in adolescents [12–15].

Adolescence is a critical transitional period, but it is also the main phase in the development and construction of Social Skills (SS) of an emotional, physical, and health profile [16–19]. Moreover, having a good repertoire of SS is a well-known protective factor for health problems. In addition, healthy habits and risk behaviors are learned early in the life cycle [20] with SS being a positive predictor of a healthy lifestyle. Thus, the lack of SS has repercussions both on physical and socio-emotional health, through the involvement in high-risk behaviors, which may lead to difficulties in adolescent development [16–19].

It is then essential to promote individual and social behaviors that can educate adolescents in their ability to make choices, especially if we consider the influence of the adolescents` lifestyle on the health and well-being in adulthood [21–23].

According to Del Prette and Del Prette [16], the family and the schools are the two main institutions and the contexts that have greater and direct responsibility for the integral development of adolescents. Additionally, the school has also a significant influence on the behavior and training of adolescents through the development of behaviors, abilities, and values influenced by peers [24, 25].

In sum, prevention and intervention in a school context should be highly prioritized in this population. Therefore, the main goals of this study are 1) to characterize Lifestyles and SS in a sample of Portuguese adolescents, and 2) to identify homogeneous subgroups (clusters) that allow the analysis of the behavioral profile of Lifestyles and SS, in order to promote a healthy lifestyle in this population, particularly in the group with the greatest need of being included in the skills-based health education programs.

## Methods

### Study design and sample

This is a cross-sectional study performed with adolescents attending schools in Portugal. Firstly, the Executive Boards of the five schools in the Tâmega and Sousa region were contacted to present the objectives and request authorization for the study. Written consent from the family was also required, as well as the authorization of the National Commission of Data Protection. A consent letter was sent home 2 weeks before the measurements. Students’ participation was voluntary, and they were asked to give their verbal informed consent. Data collection took place between February and March of 2017. The questionnaires were administered by undergraduate nursing students. Regarding the guarantee of anonymity, the students were instructed not to write their names when completing the questionnaire, placing it afterward in a box placed in the room for that purpose. At the end of the data collection, the team members assigned a code for each questionnaire, which took, on average, 30 to 40 min to be completed. This study followed the ethical principles reported in the Helsinki Declaration and was approved by the Ethical Evaluation Committee of Cooperativa de Ensino Superior Politécnico e Universitário (CESPU), Porto, Portugal.

### Instruments

A *socio-demographic questionnair*e, which included age, years of education, and school was used.

*My Lifestyle Questionnair*e (MLQ) [26]: assesses adolescents’ lifestyle and was based on the Lifestyle Assessment Questionnaire of Hettler [27]. The My Lifestyle Questionnaire is comprised of 28 items answered according to a 5-point Likert scale (1=“ *almost never*” and 5 = “*almost always*”) and contain five subscales: Physical Exercise (PE, e.g., “*I walk or cycle daily*”); Nutrition (NUT, e.g., “*I avoid eating fatty foods*”); Self-care (SFC, e.g., “*I sleep enough hours to feel rested*”); Monitored Safety (MS, e.g., “*When I travel by car in the front seat, outside the city, I wear a seat belt*); and Use of Drugs (UD, e.g., “*I don’t drink more than two alcoholic drinks a day*”). The total scale ranges between 28 and 140, with the higher result indicating a healthier lifestyle. The total scale was transformed into a scale of 0–100%. The Cronbach alpha in the original version was 0.76 for the total scale and 0.42 in Monitored Safety, 0.67 in Physical Exercise, 0.52 in Use of Drugs, 0.78 in Nutrition, and 0.67 in Self-care subscales. In the present study, the alpha for the total scale was 0.85 and 0.41 in Monitored Safety, 0.61 in Physical Exercise, 0.67 in Use of Drugs, 0.76 in Nutrition, and 0.77 in Self-care subscale.

The *Social Skills Inventory* (SSI) [28] assesses SS in adolescents and is comprised of 38 items, which evaluate the SS based on everyday situations of social interaction. Items are divided into six subscales skills: (1) Empathy (EMP, e.g., “*When I notice that a colleague is in trouble, I offer my support*”); (2) Assertiveness (ASS, e.g., “*When a person makes a request that I find improper, I refuse*”); (3) Self-control (SC, e.g., “I *react calmly when things don’t go as I wish*”); (4) Civility (CIV, e.g., “*When I’m leaving a place, I say goodbye to everyone*”); (5) Affective Approach (AA, e.g., “*When I want to date someone, I tell him/her at the first chance*”); and (6) Social Development (SLD, e.g., “*At school or in my job, I make oral presentations in groups when requested*”). The items are based on a 5-points Likert scale: 0 = “0–2 times”, 1 = “3–4 times”, 2 = “5–6 times”, 3 = “7–8 times”, 4 = “9–10 times”, regarding the number of times the adolescent presents the indicated action or reaction. A higher result indicates a better SS repertoire. To minimize the social desirability effect, 15 out of 38 items were formulated in the negative sense. Data were converted into a percentile system for both the total scale and the subscales. In each percentile category, an interpretation defined by the authors was associated: category1: percentile between 01 and 25- Below the lower average repertoire of SS; category2: percentile between 26 and 35- A lower average repertoire of SS; category3: percentile between 36 and 65- Good repertoire of SS; category4: percentile between 66 and 75- Elaborate repertoire of Ss; and finally, category5: percentile between 76 and 100- Highly elaborate repertoire of SS. The Cronbach alphas in the Brazilian adolescent version [29] were the followings: Total Scale: 0.89; Empathy: 0.82; Self-control: 0.73; Civility: 0.75; Assertiveness: 0.68; Affective Approach: 0.70; Social Development: 0.61. In this study, the alpha of the total scale was 0.95 and the alphas of the subscales were the following: Social Development: 0.64; Affective Approach: 0.73; Self-control: 0.77; Assertiveness: 0.80; Civility: 0.88; and Empathy: 0.90.

### Data analysis

Data analysis was based on descriptive, correlational, and classificatory statistics, supported by SPSS software version 24. Descriptive statistics (Mean/Standard Deviation) were used to characterize the sample and to present the descriptive values ​​of the questionnaires. The total score of the SSI was calculated only for the adolescents who answered the 38 items and, therefore, only 442 adolescents were analyzed. Regarding OMEV, 431 participants were not analyzed for the same motive. Participants excluded from the analyses were different from the participants included in the analyses regarding gender (X^2^_(1)_ = 8.631, *p* = .002) and age (X^2^_(1)_ = 15.17, *p* < .001); that is, the group of participants excluded from the analyses was composed of more male students and younger ones, when compared to the group of participants who were included in the analyses. This makes sense because the items with the highest index of missing values ​​are those dealing with sexual intercourse and substance use (see supplementary material).

Then, a Pearson correlation was performed, given the normality of the sample, in order to assess the relationship between Lifestyle and SS. To establish a profile of the participants, a Cluster Analysis (K-means) was applied considering the SSI percentiles of the total scale and subscales, as well as the total scale and subscales of the MLQ ​​(considering a 0–100 scale %). K-means is a simple clustering method used when there are unlabeled data (i.e., data without defined categories or groups). This is an iterative algorithm that tries to partition the data set into different predefined non-overlapping subgroups (clusters), where each data point belongs to only one group. It brings together intracluster data points as similar as possible, while also keeping clusters as different (as far apart) as possible. Thus, the goal of this algorithm is to find groups in the data clustered based on feature similarity. To determine the optimal number of clusters two methods were used a direct method - the elbow and silhouette - and a statistical method through a specific library of R package. A R package fpc [30] was used that allows, through bootstrap resampling, to verify if the structure of the clustering solution remains, under admissible changes to the data set [30, 31]. The optimal number of clusters depends on the method used for measuring similarities and the parameters used for partitioning. In this study, a solution of 3 clusters was adopted, being this the most adjusted to the results. The stability of the solution was tested by the Jaccard coefficient that measures the similarity or dissimilarity between two subjects. For the solution of three clusters, the average Jaccard index obtained is, for all clusters, greater than or equal to 0.95 (note: the closer to 1 the better). The analysis was performed using software R version 3.4.3. (package fpc). Kruskal-Wallis tests were used to analyze the differences in terms of sex and age. Finally, descriptive statistics (Mean and standard deviation) were mainly used to characterize the sample in cluster 2 because this was the one that most needed intervention in education and health promotion. A Pearson correlation was used to assess/evaluate the relationship between subscales and, finally, difference tests (T-tests) were employed to verify any differences in gender and age in this cluster. The level of significance was set to 0.05.

## Results

### Sample characterization

This study included 1008 adolescents attending the 7th to the 9th grade of five middle schools from the Tâmega and Sousa region of Portugal, with a mean age of 13.43 years (SD = 1.1). The majority of the adolescents belonged to the group aged between 12 and 14 years (85.2%), with the remaining 14.8% having between 15 and 17 years, homogeneously distributed by the 3 years of schooling (7th grade = 35.1%, 8th grade = 33.6% and 9th grade = 31.3%). Students (*n* = 556) who completed 98% of each questionnaire were included in the analysis.

Regarding Lifestyle, the total mean score was of 72.26% (0–100% scale), thus indicating a healthy Lifestyle. The Monitored safety subscale had a mean score of 65.69% while the subscale Nutrition had the lowest mean score, 59.97%, however still higher than 50%.

Concerning SS, the mean percentile was found to be 67.33 (corresponding to an overall mean score of 108.13). Half of the sample (50.7%) showed a highly elaborated repertoire of SS, 11% an elaborate repertoire, 20.1% a good repertoire, and 2.7% a lower average repertoire of SS. Nevertheless, 15.5% of the students presented below the lower average repertoire of SS.

### Relationship between lifestyle and social skills subscales

Positive and significant relationships were found between the MLQ and SSI subscales (Table 1), indicating that adolescents with higher SS showed a healthy Lifestyle and vice versa.

**Table 1:** Pearson’s coefficient between the subscales of lifestyle and social skills questionnaires

	1.	2.	3.	4.	5.	6.	7.	8.	9.	10.	11.
1. SSI- Empathy	1										
2. SSI- Self control	.657^**^	1									
3. SSI- Civility	.812^**^	.567^**^	1								
4. SSI- Assertiveness	.784^**^	.584^**^	.704^**^	1							
5. SSI- Affective approach	.684^**^	.524^**^	.524^**^	.594^**^	1						
6. SSI- Social development	.703^**^	.575^**^	.596^**^	.701^**^	.587^**^	1					
7. MLQ- Physical Exercise	.016	.067^*^	.052	.030	.120^**^	.195^**^	1				
8. MLQ- Nutrition	.080^*^	.157^**^	.100^**^	.069^*^	.072^*^	.104^**^	.247^**^	1			
9. MLQ- Self-care	.240^**^	.217^**^	.227^**^	.228^**^	.167^**^	.230^**^	.225^**^	.403^**^	1		
10. MLQ- Use of Drugs	.252^**^	.182^**^	.212^**^	.231^**^	.111^**^	.158^**^	.144^**^	.311^**^	.603^**^	1	
11. MLQ- Monitored safety	.123^**^	.074^*^	.119^**^	.154^**^	.078^*^	.114^**^	.153^**^	.246^**^	.337^**^	.284^**^	1

*SSI* Social Skills Inventory, *MLQ* My Lifestyle Questionnaire, Pearson coefficient; *p < .05; **p < .01

### Cluster analysis

Cluster 1, the “Adjusted” (*n* = 92), corresponded to adolescents who had a less elaborate repertoire of SS, but with good indicators in all subscales of Lifestyle. In this cluster, students from categories 1 and 2 were included, i.e., those who presented results categorized as “below the lower average” and the “lower average of social skills” (see categories of percentiles defined in instruments section).

Cluster 2, the “Sociable” (*n* = 115) comprised adolescents with a good repertoire of SS Skills (category 3 of percentile) and with good indicators of Lifestyle, namely in the Physical Exercise, Self-care and Use of Drugs scales (although not as good as those in the cluster 1). However, this group showed poor Lifestyle indicators in Nutrition and Monitored Safety subscales.

**Fig. 1:**
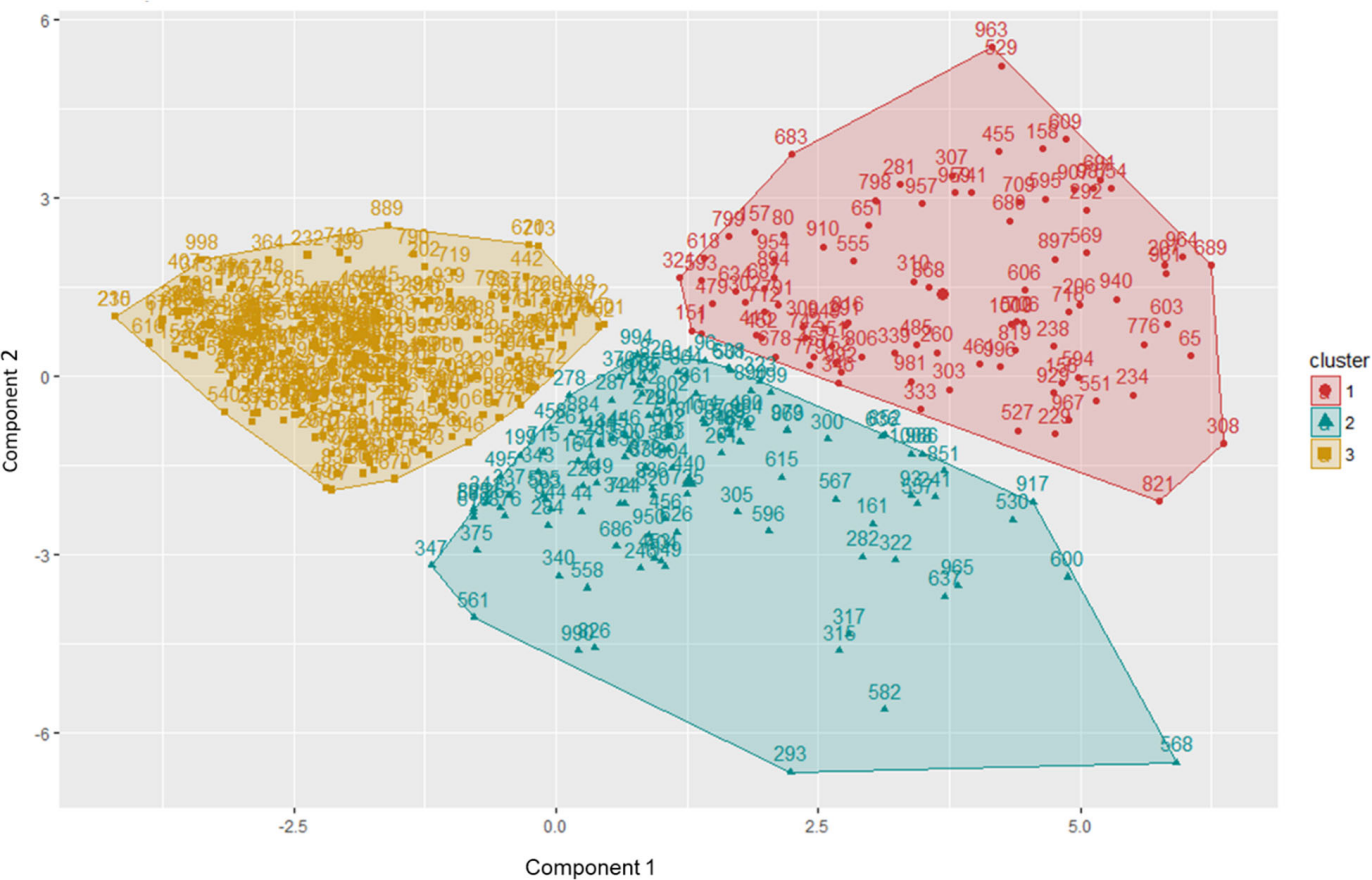
K-means Clusters. Cluster 1 (*n* = 92) presented a less elaborate Social Skills repertoire, but with good indicators in all Lifestyle subscales – it was named “*Adjusted*” cluster. Cluster 2 (*n* = 115) was formed by adolescents with good repertoire of Social Skills and good indicators of Lifestyle, but with poor Lifestyle indicators in the subscales Nutrition and Monitored Safety– it was called “*Sociable*”. Cluster 3 (*n* = 258) included adolescents with a highly elaborate repertoire of Social Skills and the best Lifestyle indicators – it was called a “*Healthy*” cluster

Cluster 3 (*n* = 258), the “Healthy”, included adolescents with an elaborate, but especially highly elaborate repertoire of SS (categories 4 and 5), and the best Lifestyle indicators (Fig. 1). In Fig. 1, observations are represented by points, through an analysis of principal components (APC), being represented according to the first two main components. Table 2 shows the sample number and the average values of the variables considered in each cluster.

**Table 2:** Sample number and the average values of the variables considered in each cluster

Cluster	n	Empathy	Self-control	Civility	Assertiveness	Affective approach	Social development	Total SSI	Physical exercise	Nutrition	Self-care	Use of drugs	Monitored safety	Total MLQ
1. Adjusted	92	20.09	32.48	23.25	27.52	39.23	29.42	21.46	66.67	60.43	71.90	72.78	67.03	68.96
2. Sociable	115	62.37	64.17	63.04	64.75	68.04	63.18	66.59	63.84	38.70	60.24	62.17	46.95	55.77
3. Healthy	258	80.33	78.46	81.82	79.18	79.69	78.15	86.25	80.98	68.68	86.50	86.56	77.71	81.81

*SSI* Social Skills Inventory, *MLQ* My Lifestyle Questionnaire

### Differences in age and gender between clusters

There were significant differences in age between clusters 1 and 2, and clusters 2 and 3 (X^2^_(2)_ = 15.18,*p* < 0.05), but there were no differences between clusters according to gender, i.e. the group of “Sociable” adolescents had a higher mean age than the groups of “Adjusted” and “Healthy” adolescents (M = 14.0, SD = 1.12).

### Sample characterization of cluster 2, the “sociable”

The group of “Sociable” adolescents included a majority of boys (61.74%, *n* = 71) with ages ranging between 12 and 14 years old (70.43%, *n* = 81). As it was possible to verify, the “Sociable” adolescents presented lower indicators in the subscales of Nutrition and Monitored Safety. By analysing these two subscales in detail (Table 3), the results showed that in the Monitored Safety subscale, item 13 was the one that presented the lowest value ​​(*MLQ13-“I do not drive (car, motorized, etc.) when I drink too much, or I do not travel with someone who drank too much”*, while in the Nutrition subscale, item 22 presented the lowest value (*MLQ22-“I avoid eating foods that are made with sugar*” (Table 3).

**Table 3:** Items descriptive measures of the Monitored Safety and Nutrition subscale

	Minimum	Mean (SD)	Maximum
**Monitored Safety items**			
MLQ .13: *I do not drive (car, motorized,* etc.*) when I drink too much, or I do not travel with someone who drank too much*	1.00	**3.43 (1.91)**	5.00
MLQ .14: *When I drive, or when traveling in some vehicle, I like to stay within speed limits*	1.00	4.06 (1.41)	5.00
MLQ .15: *When I drive in the front seat, out of town, I put the seat belt*	1.00	4.90 (0.36)	5.00
**Nutrition items**			
MLQ .4: *I am careful with what I eat so as to maintain the recommended weight for the height I have*	1.00	4.01 (0.99)	5.00
MLQ .5: *I am careful with what I eat so as to reduce salt intake*	1.00	3.75 (1.05)	5.00
MLQ .6: *I plan my diet so that it is balanced in nutrient variety*	1.00	**3.66 (1.09)**	5.00
MLQ .18: *I avoid eating fat foods*	1.00	3.98 (0.88)	5.00
MLQ .22: *I avoid eating foods that are made with sugar*	1.00	**3.45 (1.09)**	5.00

*MLQ* My Lifestyle Questionnaire, *SD* Standard Deviation

### Relationship between the nutrition and monitored safety subscales and the social skills subscales

There was a positive and significant relationship between the Nutrition and the Civility subscales (*r* = 0.21, *p* < .01). However, no significant relationships were found between the Monitored Safety and SS subscales.

### Gender and age differences in cluster 2, the “sociable”

Significant gender differences were found in the subscale Physical Exercise (*t*_(86)_ = 2.55, *p* = .013), i.e. girls do less exercise compared with boys, yet no differences were found according to age.

## Discussion

The results suggest that this sample of adolescents presents a healthy Lifestyle, assessed by the Lifestyle Questionnaire. Although higher than 50%, the Monitored Safety and Nutrition subscales were the ones displaying a lower average score. Concerning SS, half of the sample presented a highly elaborate repertoire and, as the literature indicates and it was expected, the adolescents with the healthiest Lifestyle are those with improved SS resources [32]. Besides, a solution of three clusters was found with cluster 3, the “Healthy”, being the largest one and cluster 2, the “Sociable”, the one in most need of skills-based health education programs.

Regarding the Monitored Safety subscale, the item assessing if adolescents travel with someone who drank too much was the one that presented the lowest values (M = 3.43, SD = 1.91, ranging between 1 to 5). This result may be related to the mean age of the current sample and not risk behavior. However, it is worth mentioning that in Portugal, the greatest occurrence of road accidents in motorized two-wheeled vehicles occurs mainly in male adolescents [33, 34] and, in the European Union, the highest prevalence of deaths has been observed in male adolescents between the ages of 15 and 29 years [35, 36]. Moreover, one of the main causes of death and disability is alcohol consumption [35], and the injuries related to accidents or violent behaviors frequently associated with alcohol consumption are indicated as the major cause of death in childhood and adolescence (from 5 to 19 years) [34]. Although recent data suggested a decreasing trend in the prevalence of alcohol consumption in both sexes at age 15, it also implied an increase in 16-year-old female adolescents [37], emphasizing the urgent need to address this major cause of death and disability in adolescents, especially if associated with driving behaviors. Fortunately, according to the most recent results of the Health Behaviour in School-aged Children study in Portugal [33], from 6742 adolescents, most of them have never tried tobacco (93,7%), rarely used alcohol (90%) or drugs (96.1%), and 88.2% have reported never getting drunk.

Concerning Nutrition subscale, the item evaluating the consumption of foods with sugar had the lowest average score, highlighting that an increased proportion of adolescents does not avoid foods with sugar. These results are in line with the national scenario, as demonstrated in the Health Behavior in School-aged Children study, where more than half of the Portuguese adolescents who participated in the study reported consuming sweets and soft drinks at least once a week, and more than two-thirds said that they sometimes ate unhealthy foods [38–41]. Despite the encouraging results of the present study, which highlights a healthy Lifestyle in a representative sample of adolescents in the Tâmega and Sousa region, Nutrition/food and Monitored Safety still require greater investment in the implementation of skills-based health education programs, given the association of SS with Lifestyle (ranging between r = .07 and r = .25 with *p* < .01 and *p* < .05).

Regarding the second aim of this study, the cluster analysis allowed the identification of three groups of adolescents with different behavior profiles. One of the clusters identified was named “Adjusted” because the adolescents here included showed less elaborate repertoire of SS, but good Lifestyle indicators. This group seems to have sufficient SS to adopt good and healthy behaviors. The second cluster was named “Sociable” because this group of adolescents showed high social abilities but some difficulties in adopting healthy and adequate Lifestyles, especially regarding Nutrition and Monitored Safety, suggesting a protective effect of SS [32]. The third cluster was called “Healthy” because it was the group of adolescents displaying a highly elaborate repertoire of SS and with a healthier Lifestyle.

In addition, results also showed that the Nutrition subscale was related to Civility skills, suggesting that the promotion of this social competence can, in turn, increase health awareness and health behaviors, especially with regards to diet and nutrient planning. Interestingly, no significant relationships were found between the Monitored Safety subscale and SS. This result may be related to the age range of the sample since these adolescents did not have the legal age to drive yet (> 18 years old). However, among the Monitored Safety behaviors evaluated in the questionnaire used in the study (“*When I travel by car, I put my seat belt; I did not travel with a driver who drank too much; When I travel with someone, I like to maintain speed limits*”), not traveling with a driver who drank too much was the behavior reported with less frequency. It is also noteworthy that this group of adolescents, designated as “Sociable”, was older than the group of adolescents in the “Adjusted” and “Healthy” clusters, but even so, ages ranged from 12 to 14 years old. Probably, the adolescents in this group are still too young to drive with friends. We also found that girls in this cluster practice less exercise when compared to boys, which is in agreement with the literature [9]. Knowing that levels of physical activity are below those recommended by WHO [40], this outcome emphasizes the need to promote the involvement of girls in physical activity. However, we did not find differences in Lifestyle according to age, which may be associated with the homogeneity of the sample (70% of this cluster sample consists of adolescents between 12 and 14 years old).

### Limitations and directions for future research

This study has some limitations that should be pointed out. The instruments used were in a self-report format and anthropometric data of adolescents, while demographic data of parents (such as age, socioeconomic status, and household composition) were not collected. The study included only adolescents from the Tâmega and Sousa region, requiring a careful generalization of the findings. However, it is important to highlight that this region is characterized by a high prevalence of Tuberculosis [42], which, in addition to other factors, is also related to an unhealthy lifestyle [43].

For future studies, we suggest an assessment of adolescents and their parents, given the direct influence of parents’ Lifestyle on their children’s Lifestyle [44, 45]. Moreover, White and Halliwell [46] found that adolescents’ perception of the mealtime environment contributes to the protective effect of family meals, i.e. family meals were significantly associated with a lower likelihood for alcohol and tobacco use. In addition, parental styles should be evaluated; especially the parental style of the mother, since literature has been suggesting that it influences the children’s adoption of high-risk behaviors [47]. Screen time consumption is also an important concern, given its association with a reduced level of physical activity [48], and so future studies should control for this variable. Several studies have demonstrated an advantage in the use of text messages, internet programs, and chats, apps (e.g. WhatsApp), as effective tools to instigate behavior changes in adolescents [49, 50]. This is, in fact, a controversial topic that needs further research.

### Implications for practice

This study reveals some implications for practice. Concerning the sample under study, we suggest regular health education sessions mainly focused on sugar intake behaviors, meal planning, and travel safety with a driver who drinks too much (i.e. Social Skills training), as well as promoting the involvement of girls in the practice of physical activity. Given that previous behavior is the best predictor of the intentions to adopt healthy behaviors, namely having a balanced diet, practicing sports, not drinking alcohol, and not smoking or taking drugs [51], we suggest that health education sessions should be strongly implemented in the school context [52, 53]. Nonetheless, there are several activities more attractive for girls that could also be included in the physical activity curriculum, rather than football or volleyball. The promotion of a healthy lifestyle should be included in the school curriculum and be transversal to all academic disciplines.

In general, health education sessions should include factors that protect the adoption of risk behaviors, engage the main contexts of adolescent life, and address various health behaviors and target risks [38, 54]. The school context is privileged to carry out actions of this nature, but the inclusion and involvement of both adolescents and parents become fundamental. The promotion of an available repertoire of SS that educates adolescents to a competent social style in Lifestyle choices is essential [55, 56]. Moreover, supporting emotional regulation, as well as adaptive coping strategies, is crucial for health promotion, especially in this population [57]. For example, self-regulation cognitions are positively related to healthy eating in adolescents [58]. Managing emotional regulation during class, conflict resolution, decision making, and choice may help adolescents in the adoption of healthy and protection behaviors. The most recent report of the Health Behaviour in School-aged Children study in Portugal [33] recommends the continuation of adolescent health programs, including sex education in schools, higher age limits for alcohol consumption, and obligatory use of seat belts and helmets, aiming to promote healthier lifestyles in adolescents, prevent deaths and disability.

## Conclusions

This was the first study focused on the relationship between lifestyle and SS in Portuguese adolescents, which identified adolescents at risk of having an unhealthy Lifestyle by using short questionnaires and a simple cluster analysis technique. In general, the adolescents in this sample revealed a healthy lifestyle and a highly elaborated repertoire of SS. The “Healthy” cluster was the largest in the sample and was composed of adolescents with highly elaborated SS and good indicators of a healthy lifestyle. Besides, the results emphasize that the students included in the group called “Sociable” are the ones that need to be most included in skills-based health education programs. Nutrition and Monitored Safety behaviors displayed low values and, therefore, present a greater need for awareness, sensitization, and intervention in the school context. In conclusion, the promotion of a healthy lifestyle should be part of the academic curriculum and be transversal to all academic disciplines.

## Supplementary Information

**Additional file 1.**

## Data Availability

The dataset used during the current study is available from the corresponding author by reasonable request.

## Abbreviations

LS: Lifestyle
MLQ: My Lifestyle Questionnaire
SSI: Social Skills Inventory
SS: Social Skills
EMP: Empathy
SC: Self-control
CIV: Civility
ASS: Assertiveness
AA: Affective Approach
SLD: Social Development
PE: Physical Exercise
NUT: Nutrition
SFC: Self-care
UD: Use of Drugs
MS: Monitored Safety.
