# Supplementary material for: Behavioral profile, lifestyle and social skills in Portuguese adolescents
Source: BMC Public Health. 2021 Feb 18;21:384. doi: 10.1186/s12889-021-10355-1 (PMC7893771; doi:10.1186/s12889-021-10355-1)
Supplement: Supplementary file 1 — Additional file 1. [file 12889_2021_10355_MOESM1_ESM.docx]

| **Table X**– Descriptive statistics of Social Skills Inventory | | | | | | | | |
| --- | --- | --- | --- | --- | --- | --- | --- | --- |
| Items SSI | N | | **Mean** | Median | Mode | **SD** | Min | Max |
|  | Valid | Missing |  |  |  |  |  |  |
| **1** Peço todas as informações necessárias para realizar uma tarefa | 995 | 13 | **2,74** | 3,00 | 4 | **1,30** | 0 | 4 |
| **2** Cumprimento as pessoas | 995 | 13 | **3,27** | 4,00 | 4 | **1,18** | 0 | 4 |
| **3** Agradeço quando alguém me faz um favor | 995 | 13 | **3,59** | 4,00 | 4 | **0,93** | 0 | 4 |
| **4** Despeço-me das pessoas | 993 | 15 | **3,28** | 4,00 | 4 | **1,10** | 0 | 4 |
| **5** Consigo aceitar críticas | 989 | 19 | **2,83** | 3,00 | 3 | **1,15** | 0 | 4 |
| **6** Faço pequenas coisas pelas pessoas sem que me peçam | 992 | 16 | **2,84** | 3,00 | 4 | **1,16** | 0 | 4 |
| **7** Elogio quando alguém faz algo de bom | 994 | 14 | **3,02** | 3,00 | 4 | **1,10** | 0 | 4 |
| **8** Consigo manter a calma | 989 | 19 | **2,52** | 3,00 | 4 | **1,36** | 0 | 4 |
| **9** Agradeço quando alguém me elogia | 993 | 15 | **3,44** | 4,00 | 4 | **1,02** | 0 | 4 |
| **10** Digo que quero "curtir" na primeira oportunidade | 873 | 135 | **1,82** | 2,00 | 0 | **1,55** | 0 | 4 |
| **11** Consigo tomar a iniciativa de terminar a conversa | 988 | 20 | **2,79** | 3,00 | 4 | **1,27** | 0 | 4 |
| **12** Recuso quando uma pessoa faz um pedido | 985 | 23 | **3,03** | 4,00 | 4 | **1,34** | 0 | 4 |
| **13** Quando quero entrar para um grupo arranjo maneira de me integrar | 984 | 24 | **2,68** | 3,00 | 4 | **1,30** | 0 | 4 |
| **14** Quando sou criticado, consigo responder sem perder o controlo | 989 | 19 | **2,20** | 2,00 | 3 | **1,35** | 0 | 4 |
| **15** Quando não quero curtir eu recuso, mesmo que seja muito insistente | 912 | 96 | **3,15** | 4,00 | 4 | **1,30** | 0 | 4 |
| **16** Quando não gosto de alguma coisa, digo com educação que não gostei e que não vou comprar | 987 | 21 | **3,21** | 4,00 | 4 | **1,18** | 0 | 4 |
| **17** Converso sobre sexo com os meus pais, sem problema | 918 | 90 | **1,48** | 1,00 | 0 | **1,56** | 0 | 4 |
| **18** Peço explicação sem qualquer problema quando alguém faz algo que não deve | 981 | 27 | **2,68** | 3,00 | 4 | **1,27** | 0 | 4 |
| **19** Não tenho problemas em dizer como acho que devem ser feitas as tarefas aos colegas nos trabalhos de grupo | 989 | 19 | **3,18** | 4,00 | 4 | **1,09** | 0 | 4 |
| **20** Faço apresentações orais quando solicitado na escola ou no trabalho | 990 | 18 | **2,72** | 3,00 | 4 | **1,30** | 0 | 4 |
| **21** Consigo conversar com pessoas de autoridade | 988 | 20 | **3,03** | 3,00 | 4 | **1,22** | 0 | 4 |
| **22** Consigo controlar a minha irritação quando criticam o meu comportamento | 988 | 20 | **2,68** | 3,00 | 4 | **1,26** | 0 | 4 |
| **23** Se os meus colegas me pressionam para fazer algo que acho errado, não faço | 993 | 15 | **3,01** | 3,00 | 4 | **1,23** | 0 | 4 |
| **24** Consigo guardar um segredo | 992 | 16 | **3,48** | 4,00 | 4 | **1,06** | 0 | 4 |
| **25** Faço perguntas para conhecer melhor alguém que quero ter como amigo?/a | 995 | 13 | **3,30** | 4,00 | 4 | **1,11** | 0 | 4 |
| **26** Consigo perceber os sentimentos de um amigo?/a que esteja com problemas | 996 | 12 | **3,34** | 4,00 | 4 | **1,10** | 0 | 4 |
| **27** Demonstro o meu aborrecimento quando me fazem algo que não devem | 993 | 15 | **3,04** | 3,00 | 4 | **1,17** | 0 | 4 |
| **28** Peço desculpa quando sei que fui inconveniente | 991 | 17 | **3,19** | 4,00 | 4 | **1,10** | 0 | 4 |
| **29** Consigo negociar uma solução boa quando um amigo?/a tem uma perspetiva contrária à minha | 985 | 23 | **2,87** | 3,00 | 3 | **1,09** | 0 | 4 |
| **30** Reajo com calma quando as coisas não correm como gostaria | 988 | 20 | **2,48** | 3,00 | 3 | **1,25** | 0 | 4 |
| **31** Ofereço o meu apoio a um amigo?/quando noto que está triste ou com algum problema | 987 | 21 | **3,36** | 4,00 | 4 | **1,07** | 0 | 4 |
| **32** Procuro convencer o meu?/minha parceiro?/a no uso do preservativo caso ela?/ele discorde | 665 | 343 | **3,02** | 4,00 | 4 | **1,47** | 0 | 4 |
| **33** Apresento calmamente o meu ponto de vista quando os meus pais contrariam o que penso | 978 | 30 | **2,81** | 3,00 | 4 | **1,21** | 0 | 4 |
| **34** Ofereço a minha ajuda quando um colega está com alguma dificuldade | 985 | 23 | **3,00** | 3,00 | 4 | **1,15** | 0 | 4 |
| **35** Convido pessoas para sair ou fazer alguma atividade quando quero fazer amizades | 982 | 26 | **2,61** | 3,00 | 4 | **1,29** | 0 | 4 |
| **36** Digo o que me desagrada, seja com quem for, em relação ao contacto físico | 939 | 69 | **2,76** | 3,00 | 4 | **1,28** | 0 | 4 |
| **37** Apresento-me quando quero conhecer alguém a quem não fui apresentado | 974 | 34 | **2,40** | 3,00 | 4 | **1,388** | 0 | 4 |
| **38** Consigo controlar a raiva quando o meu/minha irmão?/ã me irrita de alguma maneira | 958 | 50 | **2,00** | 2,00 | 0 | **1,489** | 0 | 4 |

| **Table Y: Descriptive statistics of My Lifestyle Questionnaire** | | | | | | | | |
| --- | --- | --- | --- | --- | --- | --- | --- | --- |
| Items MLQ | N | | **Mean** | Median | Mode | **SD** | Min | Max |
|  | Valid | Missing |  |  |  |  |  |  |
| **1** Faço exercício físico durante pelo menos 20 minutos por dia, duas vezes ou mais por semana | 1003 | 5 | **3,96** | 5,00 | 5 | 1,27 | 1 | 5 |
| **2** Ando a pé ou de bicicleta diariamente | 1003 | 5 | **3,65** | 4,00 | 5 | 1,30 | 1 | 5 |
| **3** Pratico desporto que faz suar, pelo menos duas vezes por semana | 999 | 9 | **3,96** | 5,00 | 5 | 1,36 | 1 | 5 |
| **4** Tenho cuidado com o que como, de modo a manter o peso recomendado para a altura que tenho | 1004 | 4 | **3,73** | 4,00 | 4 | 1,20 | 1 | 5 |
| **5** Tenho cuidado com o que como, de modo a reduzir a ingestão de sal | 1002 | 6 | **3,44** | 4,00 | 4 | 1,22 | 1 | 5 |
| **6** Planifico a minha dieta de modo a que ela seja equilibrada quanto à variedade de nutrientes | 996 | 12 | **3,22** | 3,00 | 4 | 1,26 | 1 | 5 |
| **7** Bebo mais de duas bebidas alcoólicas por dia (invertido) | 844 | 164 | **4,62** | 5,00 | 5 | 1,02 | 1 | 5 |
| **8** Durmo o número de horas suficientes para me sentir repousado | 1001 | 7 | **3,79** | 4,00 | 5 | 1,19 | 1 | 5 |
| **9** Mantenho as minhas vacinas em dia | 1005 | 3 | **4,80** | 5,00 | 5 | 0,71 | 1 | 5 |
| **10** Verifico anualmente a minha pressão arterial | 994 | 14 | **3,71** | 4,00 | 5 | 1,34 | 1 | 5 |
| **11** Vou ao dentista anualmente verificar o estado dos meus dentes | 999 | 9 | **4,21** | 5,00 | 5 | 1,09 | 1 | 5 |
| **12** Vou anualmente ao médico fazer um *check-up* | 974 | 34 | **3,98** | 4,00 | 5 | 1,22 | 1 | 5 |
| **13** Não guio (carro, motorizada, etc.) quando bebo demais, ou não viajo com um condutor que bebeu demais | 840 | 168 | **2,76** | 2,00 | 1 | 1,88 | 1 | 5 |
| **14** Quando guio, ou quando viajo nalgum veículo, gosto de me manter dentro dos limites de velocidade | 853 | 155 | **3,47** | 4,00 | 5 | 1,63 | 1 | 5 |
| **15** Quando viajo de carro, coloco o cinto de segurança | 995 | 13 | **4,71** | 5,00 | 5 | 0,80 | 1 | 5 |
| **16** Evito tomar medicamentos sem serem recomendados pelo médico | 993 | 15 | **4,31** | 5,00 | 5 | 1,18 | 1 | 5 |
| **17** Evito fumar | 894 | 114 | **4,34** | 5,00 | 5 | 1,34 | 1 | 5 |
| **18** Evito ingerir alimentos com gordura | 988 | 20 | **3,56** | 4,00 | 4 | 1,15 | 1 | 5 |
| **19** Devido aos efeitos potencialmente perigosos da cafeína evito tomar bebidas tais como café, chá ou coca-cola | 993 | 15 | **3,10** | 3,00 | 4 | 1,32 | 1 | 5 |
| **20** Evito utilizar estimulantes (anfetaminas ou outros) mesmo em épocas de exames | 915 | 93 | **4,07** | 5,00 | 5 | 1,46 | 1 | 5 |
| **21** Evito tomar tranquilizantes | 934 | 74 | **4,13** | 5,00 | 5 | 1,46 | 1 | 5 |
| **22** Evito ingerir alimentos que são feitos à base de açúcar | 996 | 12 | **3,08** | 3,00 | 3 | 1,22 | 1 | 5 |
| **23** Evito estar em ambientes saturados de fumo de tabaco | 988 | 20 | **4,03** | 5,00 | 5 | 1,30 | 1 | 5 |
| **24** Evito os ambientes muito ruidosos | 984 | 24 | **3,77** | 4,00 | 5 | 1,26 | 1 | 5 |
| **25** Evito os ambientes que tenham o ar poluído | 987 | 21 | **3,97** | 4,00 | 5 | 1,25 | 1 | 5 |
| **26** Evito mudar de parceiro sexual | 693 | 315 | **4,14** | 5,00 | 5 | 1,48 | 1 | 5 |
| **27** Evito ter relações sexuais com pessoas que conheço mal | 702 | 306 | **4,24** | 5,00 | 5 | 1,45 | 1 | 5 |
| **28** Devido às doenças sexuais evito ter relações sexuais sem tomar precauções | 693 | 315 | **4,22** | 5,00 | 5 | 1,46 | 1 | 5 |
